# Clinical Research: A Globalized Network

**DOI:** 10.1371/journal.pone.0115063

**Published:** 2014-12-17

**Authors:** Trevor A. Richter

**Affiliations:** Canadian Agency for Drugs and Technologies in Health, 600–865 Carling Avenue, Ottawa, ON, K1S 5S8, Canada; University of Bologna, Italy

## Abstract

Clinical research has become increasingly globalized, but the extent of globalization has not been assessed. To describe the globalization of clinical research, we used all (n = 13,208) multinational trials registered at ClinicalTrials.gov to analyzed geographic connections among individual countries. Our findings indicate that 95% (n = 185) of all countries worldwide have participated in multinational clinical research. Growth in the globalization of clinical research peaked in 2009, suggesting that the global infrastructure that supports clinical research might have reached its maximum capacity. Growth in the globalization of clinical research is attributable to increased involvement of non-traditional markets, particularly in South America and Asia. Nevertheless, Europe is the most highly interconnected geographic region (60.64% of global connections), and collectively, Europe, North America, and Asia comprise more than 85% of all global connections. Therefore, while the expansion of clinical trials into non-traditional markets has increased over the last 20 years and connects countries across the globe, traditional markets still dominate multinational clinical research, which appears to have reached a maximum global capacity.

## Introduction

Clinical trials have become increasingly globalized [1] due to the inclusion of more non-traditional locations, especially those in central and eastern Europe, Latin America, and Asia [2]–[4]. The increased globalization of clinical research has arisen for several reasons, but primarily due to the need for faster and more economically efficient studies [3], [5]. Moves towards standardizing and harmonizing clinical research practices have facilitated the rise of globalized clinical research [6], and there has been increasing pressure from the research community for commercial companies to make all clinical trial data available through publicly accessible registries [7]. ClinicalTrials.gov was launched in 2008 to implement Section 801 of the Food and Drug Administration Amendments Act of 2007 [8], and is the largest online clinical trial registry, containing data from more than 165,000 studies.

Previous studies have assessed the geographic distribution of clinical research [2], [5], but the full extent of globalization has not been assessed. The availability of software developed to analyze vast social networks, together with the large repository of data at ClinicalTrials.gov, has made it possible to analyze global connectivity among all those countries that have participated in multinational clinical research. Here, we describe a network analysis of connectivity among all multinational studies registered at ClinicalTrials.gov to provide the first comprehensive quantitative description of the globalization of clinical research.

## Methods

We accessed the ClinicalTrials.gov database from www.clinicaltrials.gov/ct2/resources/download. We extracted a total of 222,662 database records representing 123,774 unique clinical studies. We then categorized each study either as a single-nation study or a multinational study according to whether it was carried out in a single country or in multiple (>1) countries. Only data for multinational studies (n = 15,543) were included in subsequent analyses.

We compiled a list of countries involved in at least one multinational study. Each country was then assigned to one of six geographic regions (North America, South America, Africa, Europe, Asia, or Oceania) to allow data to be aggregated by region. All countries that were listed as participants in a multinational study were equally weighted irrespective of the number of sites or study participants per country.

To analyze connectivity among countries that participated in multinational studies, we conducted a network analysis using open-source software (Gephi ver. 0.8.2-beta) developed to analyze large networks such as those representing social media interactions [9]. For the network analysis, we extracted connections between countries that participated in the same study to create connections between pairs of countries, as follows. For example, if Study A was a multinational study that was carried out in country 1, 2, and 3, then three connections (unique pairings) were extracted as shown in **Table 1**. If Study B was carried out in country 2, 3, and 4, then the three connections shown in **Table 2** were extracted. We then combined all extracted connections into a single master-list of connections (e.g. see **Table 3**).

**Table 1:** Example of connections for a multinational study carried out in country 1, 2, and 3.

1–2
1–3
2–3

**Table 2:** Example of connections for a study carried out in country 2, 3, and 4.

2–3
3–4
2–4

**Table 3:** Example of a master-list that includes all extracted connections for all countries and all studies.

1–2
1–3
2–3
2–3
3–4
2–4

Where a specific pairing was represented multiple times (e.g. if countries 1 and 2 were both involved in several studies), we applied a weight to the connection that was equal to sum of the number of times the connection was represented; this reflected the strength of the connection based on the number of studies in which the countries both participated. For example, for the network representing Study A and B, the weight of all connections was 1 except for connection 2–3, which had a weight of 2 (**Table 4**).

**Table 4:** Example of weight assignments for specific pairings represented multiple times.

Connection (pairing)	Weight
1–2	1
1–3	1
2–3	2
3–4	1
2–4	1

**Figure 1:**
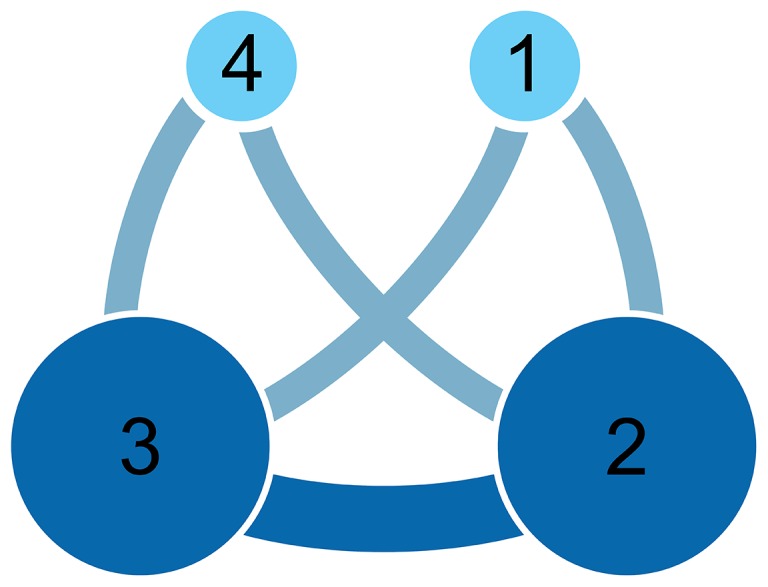
Illustration of connections within a sample network based on participation of four countries (1 through 4) in two multinational clinical trials.

We then used the weighted extracted pairs to construct a single integrated network within which all connections were represented for all studies and all countries. Therefore, the connections between countries represent direct links between countries based on participation in the same clinical studies, and the weight (thickness) of the links reflects the relative strength of the connection. Each country was represented in the network as a single node. The size of each node (area of the circle) is proportional to the weighted total number of connections for that country, thus reflecting the number of multinational studies in which each country is involved. Based on the example outlined above, the network presented in **Fig. 1** would represent the connections between countries 1 through 4 for study A and B.

The full data set and network analysis files are available for download at http://dx.doi.org/10.6084/m9.figshare.1246725.

## Results & Discussion

### Recent trends in the globalization of clinical research

Of 123,774 studies extracted from the ClinicalTrials.gov database, 109,323 (89%) studies involved only one country. The remaining 15,543 (11%) studies were multinational trials that included 185 different countries, which represents 95% of all countries worldwide (based on the US State Department recognizing 195 independent countries worldwide: www.state.gov/s/inr/rls/4250.htm#). These multinational studies were used to analyze global connectivity in a network analysis (see Methods).

**Figure 2:**
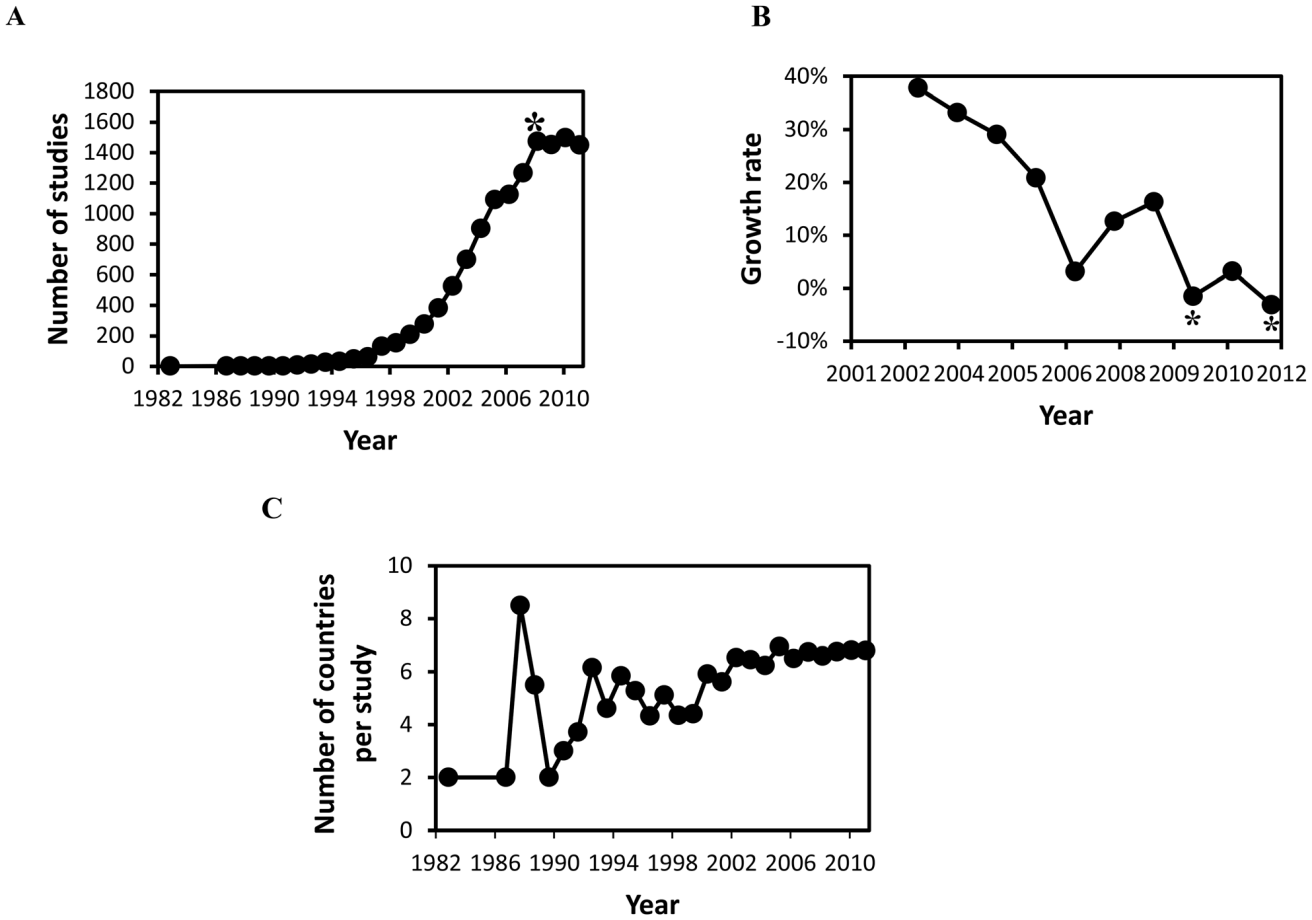
Historical trends in multinational studies registered at ClinicalTrials.gov. (*A*) Number of multinational studies per year. The asterisk (*) indicates the peak in growth in 2009. (*B*) Year-over-year growth in the number of multinational studies for the period 2003–2012. The asterisks (*) indicate years that had negative growth. (*C*) Average number of countries per multinational study for the period 1982–2012.

Each study was assigned to one year based on the study start date. Subsequent analysis of the temporal trend in the number of multinational studies initiated annually revealed explosive growth in the number of multinational studies initiated in the early 1990s, which persisted for approximately two decades (**Fig. 2**A). The number of multinational studies initiated per year peaked at 1,472 in 2009, reflecting an average annualized growth rate of 71.9% for the period 1990 through 2009. The increase in globalization peaked in 2009 (**Fig. 2**A). Indeed, **Fig. 2**B shows that the annual growth rate for the number of multinational studies initiated annually has been decreasing over the last decade, and was negative in 2010 and 2012. It would appear that since 2009, there has been a plateau in multinational clinical research. This is somewhat surprising, given the increase in the number of manufacturers who have been disclosing all available clinical trial information to registries such as ClinicalTrials.gov since 2009 [7], [10]. Nevertheless, the apparent plateau in multinational clinical research is further supported by the finding that the temporal trend in the annualized average number of countries per multinational study has remained steady at between 6 and 7 countries per study since 2003 (**Fig. 2**C). The concurrent stabilization in the growth of the number of multinational studies and the number of countries participating per study suggest that the global infrastructure that supports multinational clinical research might have reached its global capacity. Indeed, it is sobering to note 95% of all the countries in the world have already been involved to some degree in multinational studies. Several additional factors likely have contributed to the apparent stalling of growth in multinational trials, although the precise effects are difficult to determine. For instance, the increasingly stringent regulatory requirements related to conducting clinical research might have made it more difficult for sites outside traditional, developed markets to continue to meet the standards of good clinical practices (GCP) required to participate in multinational trials led by the USA and Europe. Another potential factor that might have a role in the reduction in the growth rate of multinational trials is the development of new technologies, including molecular techniques, robotics, and point-of-care technologies. These technologies are frequently costly and require specialized expertise, both of which likely will result in the inclusion of fewer investigational sites in under-developed countries, and potentially in a reduced number of traditional later-phase (phase 3 and 4) clinical trials overall.

### Geographic distribution of multinational studies

**Figure 3:**
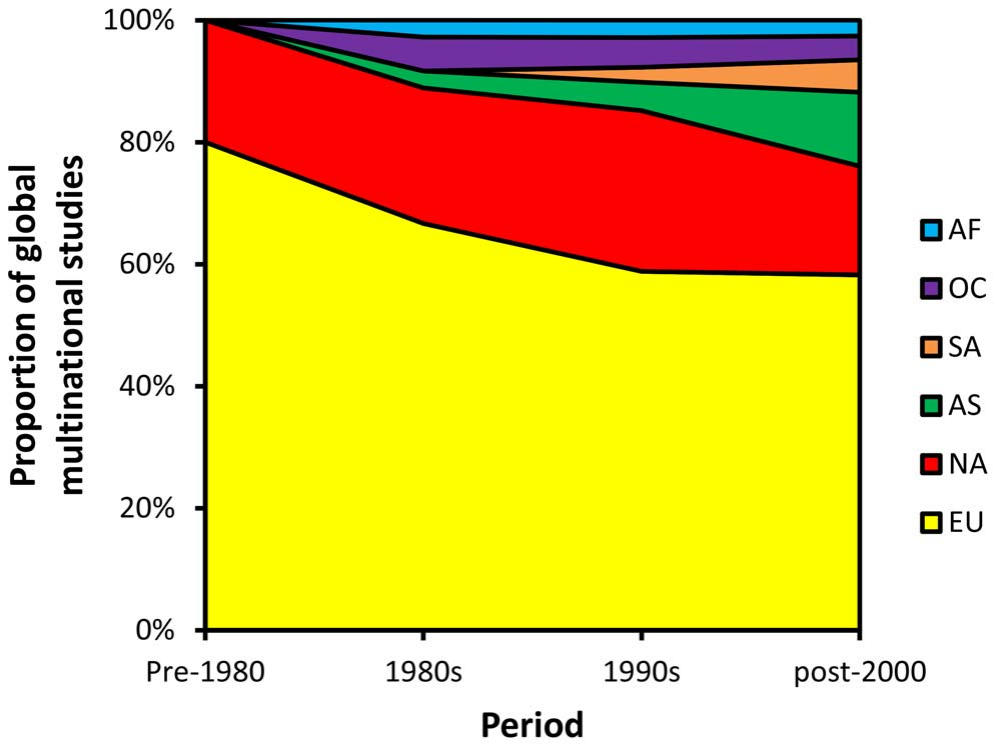
Regional distribution of the proportion of all multinational clinical studies. AF, Africa. OC, Oceania. SA, South America. AS, Asia. NA, North America. EU, Europe.

**Figure 4:**
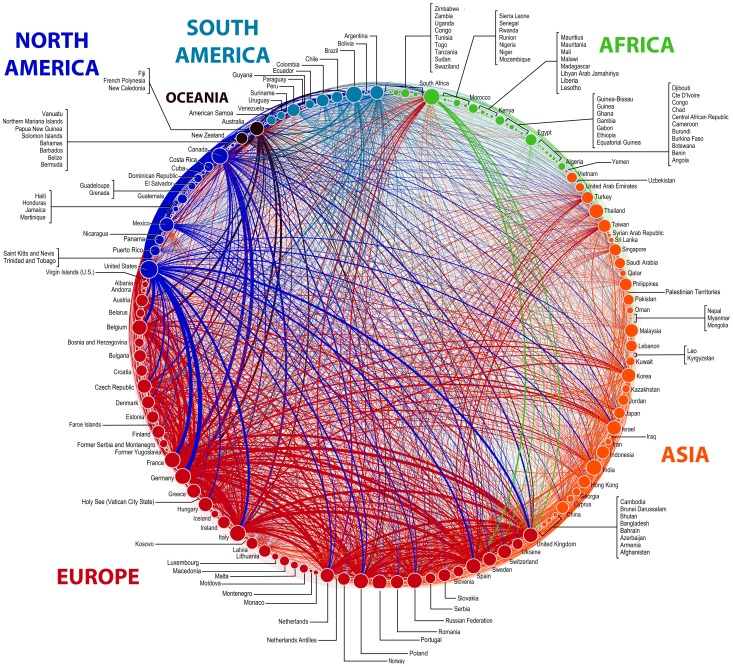
Global connectivity of countries involved in multinational clinical studies. Lines represent connections between countries that reflect participation in the same study. The thickness of the lines is proportional to the total number of connections between those countries. Each dot (node) corresponds to a country that has participated in a multinational study. The size of the nodes is proportional to the total number of multinational studies. Only countries that participated in at least 1 multinational clinical trial were included in the network.

Comparison of the proportion of global multinational trials conducted within different geographic regions revealed that the involvement of non-traditional markets in clinical research has increased since the 1980s (**Fig. 3**). Of note, over the last 20 years (comparing data for the 1990s to all data post-2000), the proportion of multinational trials that include South American countries has doubled (from 2.5% to 5.3%) and the proportion for Asia has almost tripled (from 4.7% to 12.1%) (**Fig. 3** and **4**). This finding supports the contention that growth in clinical research globally is at least partly attributable to the growth of non-traditional markets outside North America and Europe.

Ranking of individual countries revealed that the USA has been involved in more multinational studies than all other countries (n = 9,380 or 9.5% of all studies). Although this reflects the dominance of the USA in multinational clinical research, which has been described previously for clinical research in general (i.e. not limited to multinational research [1], [2]), it is possible that this finding may be exaggerated in favor of the USA due to reporting bias, because ClinicalTrials.gov is a US-based registry. While the USA was dominant as an individual country, Europe was the geographic region that dominated globally in terms of participation in multinational trials (at least one European country has participated in 58.1% of all multinational studies), followed by North America (18.5%), Asia (11.8%), South America (5.3%), Oceania (3.9%), and Africa (2.6%). North America and Europe collectively were involved in 76.5% of all multinational studies, reflecting the historical dominance of these traditional markets over the non-traditional markets in Asia and South America.

### Global connectivity through clinical research

The global network of connections based on participation in multinational clinical studies is presented in **Fig. 4**, which illustrates extensive connectivity within and between all geographic regions. Network analysis of the connections between countries based on participation in common clinical studies revealed a total of 83,887 global connections among 185 countries. The weighting of connections between countries illustrated in **Fig. 4** reflects the range in the relative strength of connections, such that some connections between countries are clearly stronger than others. For instance, the connections between the USA and several other countries appear to be among the strongest globally (**Fig. 4**). The strongest connection worldwide was that between the USA and Canada (**Fig. 4**). This strong connection obviously reflects a large number of multinational studies that include sites in both the USA and Canada (n = 4,112), but also illustrates how such empirically derived bonds between countries (based only on mutual participation in clinical studies) provide a graphic reflection of close cultural and economic ties between nations. Note that connections were stronger among countries within close geographic proximity (**Fig. 4**).

**Figure 5:**
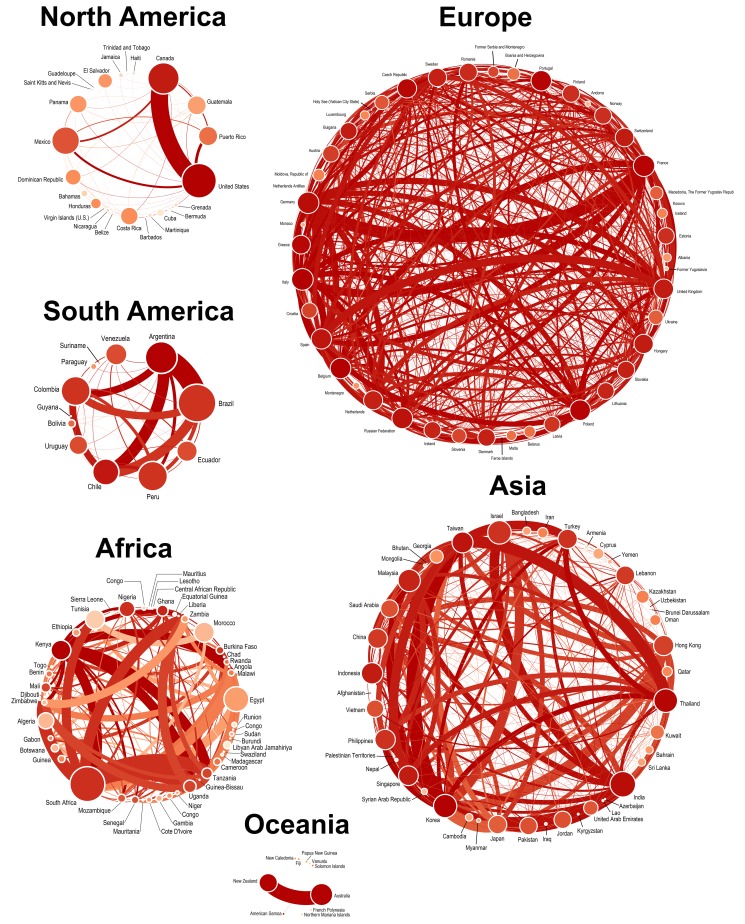
Regional connectivity of countries involved in multinational clinical studies. Conventions are as described for *Fig. 4*, with the thickness of lines being proportional to the number of connections connecting pairs of countries.

Network analysis revealed a high degree of connectivity between countries from different geographic regions. Networks for each of the six geographic regions are presented in disaggregated form in **Fig. 5**. Overall, 33.8% of all connections included countries from different regions. Europe has the highest number of connections (50,865; 60.64%), followed by North America (12,369; 14.74%), Asia (9,593; 11.44%), South America (5,137; 6.12%), Oceania (3,997; 4.76%), and Africa (1,926; 2.30%). Collectively, Europe, North America, and Asia comprised more than 85% of global connections, indicating that multinational studies have been carried out predominantly in traditional markets. This suggests that while the expansion of clinical trials into non-traditional markets has increased over the last 20 years, the traditional markets clearly dominate global clinical research [1]–[6].

The country with the most connections was Canada (5,716; 6.81%), followed by Germany (4,956; 5.91%), France (4,379; 5.22%), USA (4,116; 4.91%), and Italy (3,716; 4.43%). Nevertheless, it is interesting to note that even very small and geographically isolated countries were global connected through participation in multinational clinical trials, such as the Solomon Islands (**Fig. 5**). Furthermore, it was surprising to find that countries with severe economic or political challenges were included in the global network of connectivity, such as the Occupied Palestinian Territories, Bosnia and Herzegovina, and Congo (**Fig. 5**). While each of these observations further illustrates the truly global nature of clinical research, it should be noted that the connectivity is based on all available data that span several decades, and therefore **Fig. 5** does not show the current state of connections, but rather presents a composite historical and current perspective of global connectivity.

When the distribution of multinational clinical trials is examined after being stratified according to the condition or disease that was being studied in each trial, the global connectivity among regions noted above is further illustrated by the fact that the same conditions have been the subject of clinical trials across different regions (**Table 5**). Specifically, trials that have studied diabetes have dominated clinical research efforts in five of the six geographic regions (18% to 33% of trials per region; see **Table 5**), while breast cancer and rheumatoid arthritis are also among the five most frequently studied conditions across all geographic regions (**Table 5**). Despite this apparent homogeneity in the types of conditions that have been studied through multinational clinical trials, there do appear to be some region-specific conditions that have been studied through multinational trials, such as HIV in the Americas and Africa, lung cancer in Asia, alcoholism in Europe, and malaria in Africa (**Table 5**). Future analyses of the clinicaltrials.gov database should provide insights into temporal trends in the distribution of multinational research effort in specific therapeutic areas.

**Table 5:** Regional distribution of multinational clinical trials stratified by condition (disease).

North America	Europe	Asia	South America	Africa	Oceania
Diabetes (22.2%)	Diabetes (23.0%)	Diabetes (33.4%)	Diabetes (25.3%)	HIV (31.0%)	Diabetes (22.4%)
HIV (14.0%)	Rheumatoid Arthritis (10.6%)	Breast Cancer (10.5%)	Breast Cancer (12.7%)	Diabetes (18.3%)	Breast Cancer (11.8%)
Breast Cancer (8.2%)	Breast Cancer (9.6%)	Rheumatoid Arthritis (7.9%)	Rheumatoid Arthritis (12.7%)	Malaria (8.6%)	Rheumatoid Arthritis (10.9%)
Rheumatoid Arthritis (7.9%)	Asthma (7.1%)	Lung Cancer (7.4%)	HIV (9.7%)	Breast Cancer (7.5%)	Leukemia (8.1%)
Alcoholism (6.1%)	Alcoholism (5.6%)	Schizophrenia (6.7%)	Asthma (9.0%)	Rheumatoid Arthritis/Asthma (5.1%)	Asthma (6.4%)

The top 5 conditions are presented for each region, ranked from highest to lowest based on the number of clinical trials per condition as a proportion (%) of the total number of multinational trials for that region.

## Conclusions

The increased globalization of clinical research over the last two decades is attributable in part to increased expansion of clinical trials into non-traditional markets. This has created a geographically integrated network within which traditional markets have dominated global clinical research. However, the expansion of multinational clinical research peaked in 2009, which could reflect that the large-scale expansion of multinational clinical research effort has reached its global capacity.

The apparent stabilization of the global expansion of clinical research reflects maturation of global connectivity through clinical research. Indeed, the involvement of almost all countries in multinational research reflects the well-developed global clinical research network that spans the globe. Despite the fact that high-income countries in traditional markets have dominated multinational clinical research, the increased involvement of non-traditional markets in multinational research should allay any fear of exclusion of middle- and low-income countries from participating in clinical research and has attendant benefits. A major potential benefit of the expansion of clinical trials beyond traditional markets is the increased relevance of research to a greater number of countries, which could potentially influence clinical practice within regions that participate in multinational trials. Moreover, the increased participation of non-traditional markets in multinational research may enhance clinical practice through increased capacity development and investment by enhancing technical expertise at participating sites, providing economic incentives to improve practice standards, and providing novel technologies. On the other hand, high-income countries could benefit from the globalization of clinical research if middle- and low-income regions contribute to trial methodology research efforts. While global research has focused on conditions (e.g., type II diabetes) that are of importance primarily to high-income countries that have the economic ability to direct multinational research, the establishment of a global network of clinical research participation has also allowed for multinational research effort focused on more regionally relevant conditions, such as malaria in Africa.

Studies such as this are possible only if there are sufficient data recorded and reported internationally for clinical trials. ClinicalTrials.gov, administered by the United States National Library of Medicine, was launched in September 2008 and is the oldest and largest online registry for clinical trials. Worldwide, the number of clinical trial registries is growing and currently includes (amongst others) the European Clinical Trials Database (EudraCT: eudract.ema.europa.eu), the UK-based Current Controlled Trials (www.controlled-trials.com), and the Japan Pharmaceutical Information Center (www.clinicaltrials.jp). While the amount of data available from sources other than ClinicalTrials.gov is relatively small at present, it is anticipated that the expansion of these databases as well as ClinicalTrials.gov will facilitate valuable insights into global clinical research trends in future.
